# The Medical Association

**Published:** 1894-04

**Authors:** 


**The Medical Association.**—Our worthy contemporary *The North Carolina Medical Journal*, in the February issue, has the following timely suggestion, which we fully endorse.

"In the first place, is it worth while to be a member of the State Society? Is it worth a man's while to absent himself from his practice for four or five days each year to attend the meetings? Does it pay him to do this besides spending the money necessary for the trip? To all these questions we answer most emphatically, Yes. It is worth to any physician many times more than it costs him. Not only in the benefit he derives from respiting his tired mind and body from the daily routine of a laborious practice, from the making of firm and lasting friendships among his professional brethren, from the new ideas he catches in the discussions upon many and varied subjects, or from the Society honors to which he may attain; but the time has come when the very fact of being a member of the State Society is a matter of vast importance to the reputable physicians of our State, for the laity have accustomed themselves to judge, in some degree, a physician's standing by that fact. Often have we had the question asked us concerning different physicians: "Is he a member of the State Society?" And when answered in the negative, it was always apparent that, in the interrogator's opinion, that physician did not occupy quite so high a place, even when our reply was guarded by the assertion that there were many excellent physicians in the State who had not identified themselves with the Society. They feel, and in great measure rightly, that a physician who is not a member of the Society, does not show a proper interest in his profession, or sufficient zeal in keeping abreast of the advances that are so rapidly developing."

---

**The Austin** District Medical Society held its regular quarterly meeting March 22d ult., with a fair attendance. The papers read will appear in the JOURNAL as opportunity to use them presents. Dr. Fannie Leake read a paper entitled "The Doctors Themselves," in which she showed up some of the foibles of the doctor, and described certain evils which beset him, the thing principally complained of being the want of reciprocity on the part of the druggist towards the doctor who sends him prescriptions; and the chronic complaint of inability to make people pay doctor's bills. The paper elicited an animated discussion.

---

**The Railroad Surgeons** will have a big convention at Galveston on 9th and 10th next month, a very large attendance being expected. They will excurt to Austin and run out and take a look at our superb dam and lake.

